# *Bacteroides fragilis* Enterotoxin Induces Sulfiredoxin-1 Expression in Intestinal Epithelial Cell Lines Through a Mitogen-Activated Protein Kinases- and Nrf2-Dependent Pathway, Leading to the Suppression of Apoptosis

**DOI:** 10.3390/ijms21155383

**Published:** 2020-07-29

**Authors:** Jong Ik Jeon, Jun Ho Choi, Keun Hwa Lee, Jung Mogg Kim

**Affiliations:** 1 Department of Microbiology, Hanyang University College of Medicine, Seoul 04763, Korea; siela@hanmail.net (J.I.J.); micro-001@hanmail.net (J.H.C.); 2 Department of Biomedical Science, Hanyang University College of Medicine and Graduate School of Biomedical Science and Engineering, Seoul 04763, Korea

**Keywords:** *Bacteroides fragilis*, enterotoxin, intestinal epithelial cells, sulfiredoxin-1

## Abstract

Enterotoxigenic *Bacteroides fragilis* is a causative agent of colitis and secrets enterotoxin (BFT), leading to the disease. Sulfiredoxin (Srx)-1 serves to protect from oxidative damages. Although BFT can generate reactive oxygen species in intestinal epithelial cells (IECs), no Srx-1 expression has been reported in ETBF infection. In this study, we explored the effects of ETBF-produced BFT on Srx-1 induction in IECs. Treatment of IECs with BFT resulted in increased expression of Srx-1 in a time-dependent manner. BFT treatment also activated transcriptional signals including Nrf2, AP-1 and NF-κB, and the Srx-1 induction was dependent on the activation of Nrf2 signals. Nrf2 activation was assessed using immunoblot and Nrf2-DNA binding activity and the specificity was confirmed by supershift and competition assays. Suppression of NF-κB or AP-1 signals did not affect the upregulation of Srx-1 expression. Nrf2-dependent Srx-1 expression was associated with the activation of p38 mitogen-activated protein kinases (MAPKs) in IECs. Furthermore, suppression of Srx-1 significantly enhanced apoptosis while overexpression of Srx-1 significantly attenuated apoptosis during exposure to BFT. These results imply that a signaling cascade involving p38 and Nrf2 is essential for Srx-1 upregulation in IECs stimulated with BFT. Following this upregulation, Srx-1 may control the apoptosis in BFT-exposed IECs.

## 1. Introduction

Toxigenic *Bacteroides fragilis* (ETBF) is known to be highly related to several colonic illnesses, including colitis, inflammatory bowel diseases and colon cancers [1,2,3,4]. The *B. fragilis* enterotoxin (BFT) is the only virulence factor and is known to be the cause of the above diseases [5,6]. In addition, BFT-exposed intestinal epithelial cells (IECs) provoke inflammatory signals in the gut [1,4,7,8]. Although inflammatory signals derived from IECs are observed within the first few hours after exposure to ETBF-derived BFT, little information is available on the signals that control the fate of IECs after the inflammatory signal decreases.

Peroxiredoxins (Prxs) can reduce H_2_O_2_ and alkyl hydroperoxides and are associated with many cellular functions including protection against oxidative stress [9]. In mammalian cells, there are several Prx isoforms [10]. The Prx I–IV are inactivated through hyperoxidation of cysteine to sulfinic acid during catalysis and are reactivated through a reaction catalyzed by sulfiredoxin-1 (Srx-1) [9,11]. The promoter regions of the human Srx-1 gene have several potential binding sites for transcription factors such as NF-κB, AP-1, and Nrf2. Therefore, Srx-1 can be activated when transcriptional factors bind to the promoter in Srx-1. Several stimuli or chemicals can induce Srx-1 expression. For example, 12-O-tetradecanoylphorbol 13-acetate and lipopolysaccharide (LPS) can induce Srx-1 expression [12,13]. Nrf2 activators such as sulforaphane, tert-butylhydroquinone (tBHQ), and 3-H-1,2-dithiole-2-thione (D3 T) can induce Srx-1 expression in cultured rat cortical neurons and glial cells [14]. Ethanol treatment also enhances the Srx-1 induction in the liver of mice [15].

The suppression of Srx-1 activity may be associated with oxidative damage [12,13]. In addition, the enhanced expression of Srx-1 results in cellular proliferation and carcinogenesis [9,11,16]. Upregulated Srx-1 expression can also lead to the protection of cells from immunopathogenesis or stress damage. For example, Srx-1 prevents from LPS-induced endotoxic shock in mice [17]. Concerning oxidative stress, BFT prompts the generation of reactive oxygen species (ROS) in IECs [18] and dendritic cells [19]. Therefore, increased ROS levels may lead to enhanced antioxidant capacity as an adaptation to oxidative stress. These results led us to establish the hypothesis that the Srx-1 induction modulates the fate of IECs under the BFT-exposed condition. However, there have been no reports of the Srx-1 induction in ETBF infection.

The expression of Srx-1 is controlled by several transcriptional factors [20]. Transcription factors such as NF-E2-related factor 2 (Nrf2) and activator protein-1 (AP-1) are necessary for Srx-1 induction in LPS-treated murine macrophages [21]. In addition, a transcriptional factor nuclear factor-kappaB (NF-κB) modulates the Srx-1 expression in human hepatocellular carcinoma HepG2 cells [22]. These transcriptional factors are activated in several types of cells exposed to BFT [4,7,19,23,24]. Nevertheless, there have been no reports that the BFT-related activating signals may regulate the Srx-1 expression. Therefore, we explored the expression of Srx-1 in BFT-exposed IECs. Here we show that the p38 mitogen-activated protein kinases (MAPKs)-Nrf2 signals are essential for Srx-1 induction in IECs following exposure to BFT.

## 2. Results

### 2.1. Upregulation of Srx-1 in BFT-Stimulated IECs

**Figure 1:**
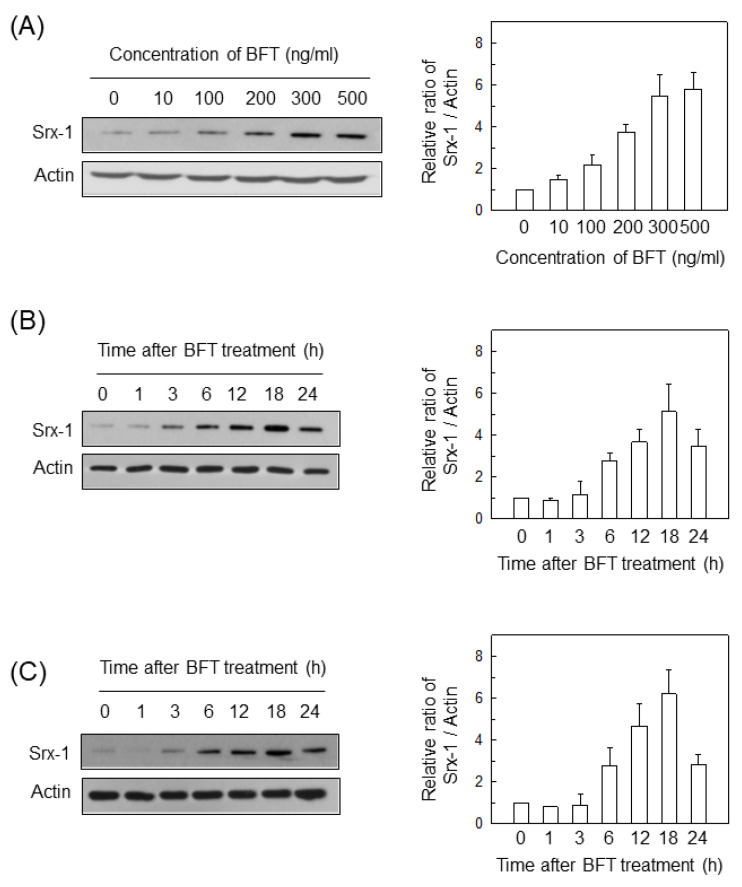
Expression of Srx-1 proteins in intestinal epithelial cells (IECs) treated with enterotoxin (BFT). (**A**) HCT 116 cells were treated with the indicated concentrations of BFT for 18 h. Protein expression of Srx-1 and actin was evaluated by western blot; (**B**,**C**) BFT (300 ng/mL) was added to HCT 116 cells (**B**) or primary intestinal CCD 841 CoN cells (**C**) for the indicated periods of time. Protein expression of Srx-1 and actin was examined by western blot. All images in (**A**–**C**) are representative of more than three independent experiments. Right panels show densitometric analysis for expressed proteins. Values represent the relative densities of each protein compared with actin (mean ± SD, *n* = 3).

The magnitude of Srx-1 protein expression was dependent on the concentration of BFT (Figure 1A). Based on these results, 300-ng/mL BFT was used in subsequent experiments. When BFT was added to HCT 116 cells, Srx-1 expression was increased in a time-dependent manner (Figure 1B). Srx-1 expression peaked approximately 12 to 18 h posttreatment. Similar results were detected in primary normal epithelial CCD 841 CoN cells (Figure 1C).

### 2.2. NF-κB and AP-1 Are Not Related with the Srx-1 Induction in IECs Stimulated with BFT

**Figure 2:**
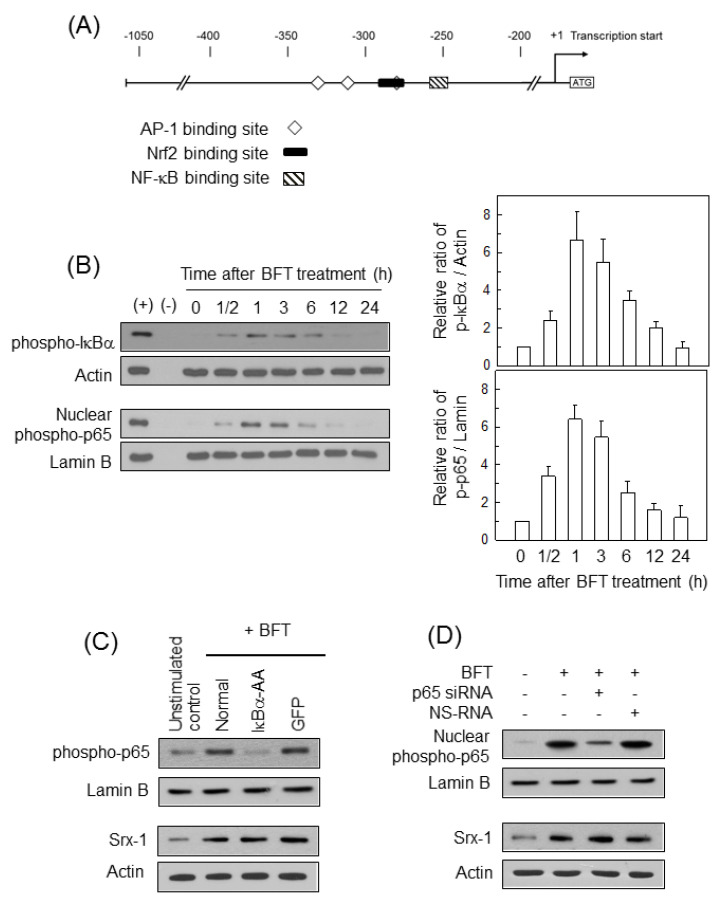
Effects of NF-κB-suppression on Srx-1 expression in BFT-exposed HCT 116 cells. (**A**) Schematic representation of human Srx gene promoter and binding sited for potential transcription factors. JASPER 2020 (http://jaspar.genereg.net/) was used to identify binding sites for NF-κB, AP-1 and Nrf2 present in the human SRX-1 promoter. Numbers indicate nucleotide position relative to the translation start site (ATG); (**B**) BFT (300 ng/mL) was added to HCT 116 cells for the indicated periods of time. In the top panels, protein expression of phospho-IκBα and actin in whole cell lysates was examined by western blot. In the bottom panels, protein expression of phospho-p65 and lamin B in nuclear extracts was also evaluated by western blot. Right panels show densitometric analysis for expressed proteins. Positive controls (+) were obtained from cells that were treated with 20 µM of TNF-α for 1 h. Values represent the relative densities of each protein compared with actin or lamin B (mean ± SD, *n* = 3); (**C**) HCT 116 cells were transfected with either lentivirus containing an IκBα-superrepressor (IκBα-AA) or control virus (GFP). BFT (300 ng/mL) was added to each group for 1 h. Protein expression of nuclear phospho-p65 was examined by western blot (top panel). In the bottom panels, BFT (300 ng/mL) was added to each group for 18 h. Protein expression of Srx-1 and actin was evaluated by western blot; (**D**) HCT 116 cells were transfected with siRNA against NF-κB p65 or NS-RNA. BFT (300 ng/mL) was added to each group for 1 h. Protein expression of nuclear phospho-p65 was examined by western blot (top panel). In the bottom panels, BFT (300 ng/mL) was added to each group for 18 h. Protein expression of Srx-1 and actin was also evaluated by western blot. All images in (**B**–**D**) are representative of more than three independent experiments.

Since the promoter region of the human Srx-1 gene has binding sites for several transcriptional factors (Figure 2A), we first asked whether activation of NF-κB may be related to Srx-1 expression in BFT-exposed IECs. HCT 116 cells increased both protein levels of cytoplasmic phospho-IκBα and nuclear phospho-p65 expression (Figure 2B). Since the activation of Nrf2 peaked approximately 3 to 6 h posttreatment in the present study and our previous results [19,25], we set the BFT stimulation time to 6 h in subsequent experiments. We next determined whether suppression of NF-κB activity may influence the induction of Srx-1 in BFT-stimulated IECs. Transfection with lentivirus containing NF-κB superrepressor (IκBα-AA) decreased nuclear phospho-p65 expression to levels similar to the unstimulated control. In contrast, the transfection with the control virus (GFP) did not affect phospho-p65 expression (Figure 2C, top panels). In this experimental model, the protein expression of Srx-1 in IκBα-AA-transfected cells did not differ from that in untransfected cells under BFT-treated conditions (Figure 2C, bottom panels). In another experiment, the p65 siRNA reduced the nuclear expression of phospho-p65 in BFT-treated HCT 116 cells (Figure 2D, top panels). In addition, suppression of NF-κB activity with p65 siRNA was not related to a change in BFT-induced Srx-1 expression (Figure 2D, bottom panels).

**Figure 3:**
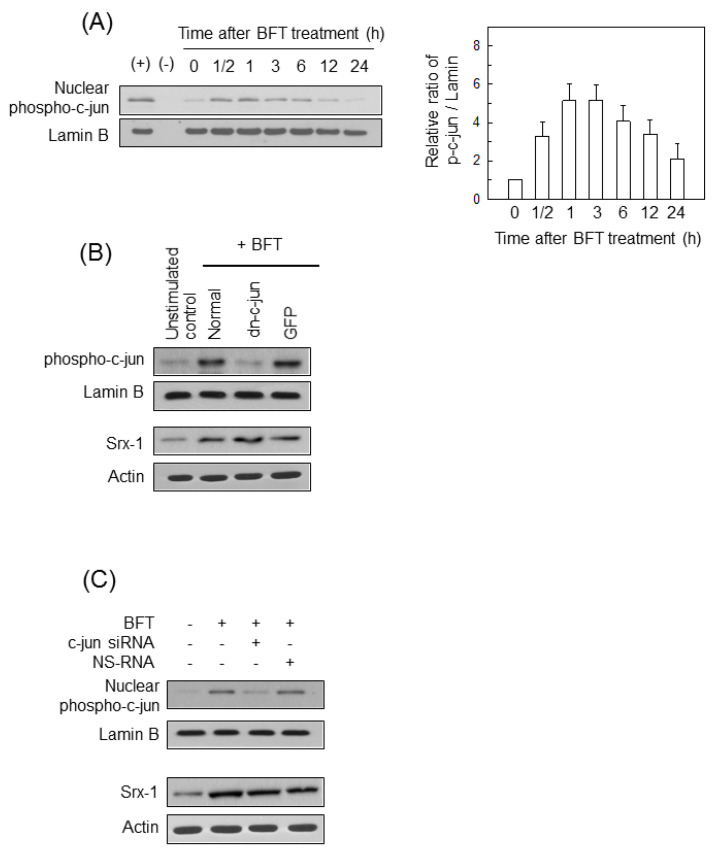
Effects of AP-1-inhibition on Srx-1 expression in BFT-exposed HCT 116 cells. (**A**) BFT (300 ng/mL) was added to HCT 116 cells for the indicated periods of time. Nuclear protein expression of phospho-c-jun and lamin B was examined by western blot. Positive controls (+) were obtained from cells that were treated with 20 µM of TNF-α for 1 h. Right panel shows densitometric analysis for expressed proteins. Values represent the relative densities of each protein compared with lamin B (mean ± SD, *n* = 3); (**B**) HCT 116 cells were transfected with either lentivirus containing a dominant-negative c-jun plasmid (dn-c-jun) or control virus (GFP). BFT (300 ng/mL) was added to each group for 1 h (phospho-c-jun) or 18 h (Srx-1). Nuclear protein expression of phospho-c-jun and lamin B was examined by western blot. Concurrently, protein expression of Srx-1 and actin was also evaluated by western blot; (**C**) HCT 116 cells were transfected with siRNA against c-jun or NS-RNA. BFT (300 ng/mL) was added to each group for 1 h (phospho-c-jun, top panels) or 18 h (Srx-1, bottom panels). Analysis of protein expression was identical to (**B**). All images in (**A**–**C**) are representative of more than three independent experiments.

We next assessed the relationship between AP-1 activation and Srx-1 induction. BFT treatment increased protein levels of nuclear phospho-c-jun expression in HCT 116 cells (Figure 3A). Based on these results, we examined if suppression of AP-1 activity may influence the Srx-1 induction. Lentiviruses containing dominant-negative c-jun plasmid (dn-c-jun) reduced phospho-c-jun expression to control levels in BFT-exposed HCT 116 cells (Figure 3B, top panels). However, control lentiviruses did not affect phospho-c-jun expression. In this model, no difference in Srx-1 expression was noted between dn-c-jun-transfected and untransfected cells (Figure 3B, bottom panels). In another experiment, the siRNA against c-jun reduced the expression of phospho-c-jun in HCT 116 cells (Figure 3C, top panels). In contrast, the c-jun siRNA against did not affect Srx-1 expression in BFT-treated cells (Figure 3C, bottom panels). These results imply that activation of NF-κB and AP-1 signals may not be connected with the upregulation of Srx-1 in BFT-exposed IECs.

### 2.3. Activation of Nrf2 Is Associated with BFT-Induced Upregulation of Srx-1 Expression in IECs

**Figure 4:**
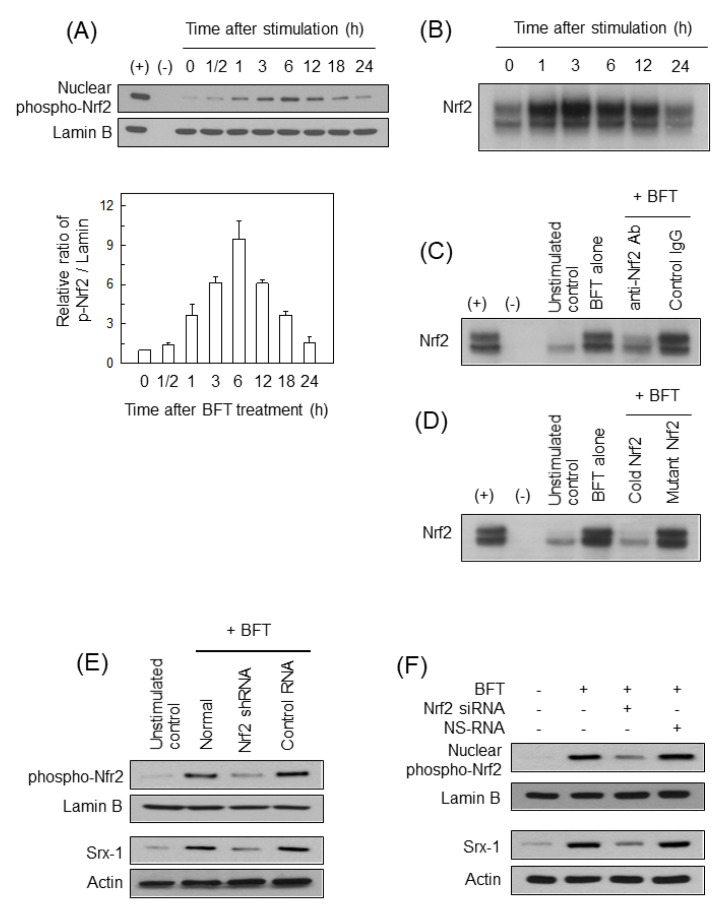
Effects of Nrf2-inhibition on Srx-1-expression in BFT-exposed HCT 116 cells. (**A**) BFT (300 ng/mL) was added to HCT 116 cells for the indicated periods of time. Nuclear protein expression of phospho-Nrf2 and lamin B was examined by western blot. Positive controls (+) were obtained from cells that were treated with 20 µM of sulforaphane for 2 h. Bottom panel shows densitometric analysis for expressed proteins. Values represent the relative densities of each protein compared with lamin B (mean ± SD, *n* = 3); (**B**) BFT (300 ng/mL) was added to HCT 116 cells for the indicated periods. Nrf2 DNA binding activity was assessed by EMSA; (**C**) HCT 116 cells were treated with BFT (300 ng/mL) for 6 h and nuclear extracts were obtained. Supershift assays using nuclear extracts were performed using anti-Nrf2 Ab and IgG isotype control Ab; (**D**) HCT 116 cells were treated with BFT (300 ng/mL) for 6 h. Nuclear extracts were added a 100-fold excess of the unlabeled probe (cold probe) or a mutant probe to the reaction, after which radiolabeled probes were added. After then, EMSA was performed identical to (**B**); (**E**) HCT 116 cells were transfected with shRNA against Nrf2 or control RNA. BFT (300 ng/mL) was added to each group for 6 h (phospho-Nrf2, top panels) or 18 h (Srx-1, bottom panels). Nuclear protein expression of phospho-Nrf2 and lamin B was determined by western blot. Concurrently, protein-expression of Srx-1 and actin was also evaluated by western blot; (**F**) HCT 116 cells were transfected with siRNA against Nrf2 or NS-RNA. BFT (300 ng/mL) was added to each group for 6 h (phospho- Nrf2, top panels) or 18 h (Srx-1, bottom panels). Analysis of protein-expression was identical to (**E**). All images are representative of more than three independent experiments.

We next examined if BFT-induced Nrf2 signaling may be related to Srx-1 upregulation. HCT 116 cells increased protein levels of nuclear phospho-Nrf2 expression (Figure 4A). In another experiment, BFT enhanced the DNA-binding activity of Nrf2 in HCT 116 cells (Figure 4B). We evaluated the specificity of Nrf2-DNA binding in BFT-exposed cells. In the supershift assay, treatment with anti-Nrf2 Ab suppressed the Nrf2-DNA binding in nuclear extracts from HCT 116 cells (Figure 4C). In the competition assay, the addition of excess Nrf2 oligomer (cold Nrf2) attenuated Nrf2-DNA binding. However, the addition of mutant Nrf2 did not change the Nrf2-DNA binding under the BFT-exposed condition (Figure 4D). Based on these findings, we assessed if suppression of Nrf2 activity may affect Srx-1 expression in cells exposed to BFT. For this experiment, a lentivirus-based shRNA system was used. Transfection of HCT 116 cells with lentivirus containing Nrf2 shRNA resulted in suppressing phospho-Nrf2 expression to the control level (Figure 4E, top panels). In addition, increased Srx-1 expression was downregulated in cells transfected with Nrf2 shRNA compared with untransfected cells (Figure 4E, bottom panels). An additional experiment also revealed that the transfection of HCT 116 cells with Nrf2 siRNA reduced both phospho-Nrf2 (Figure 4F, top panels) and Srx-1 (Figure 4F, bottom panels) signals compared with untransfected cells under BFT-exposed conditions.

**Figure 5:**
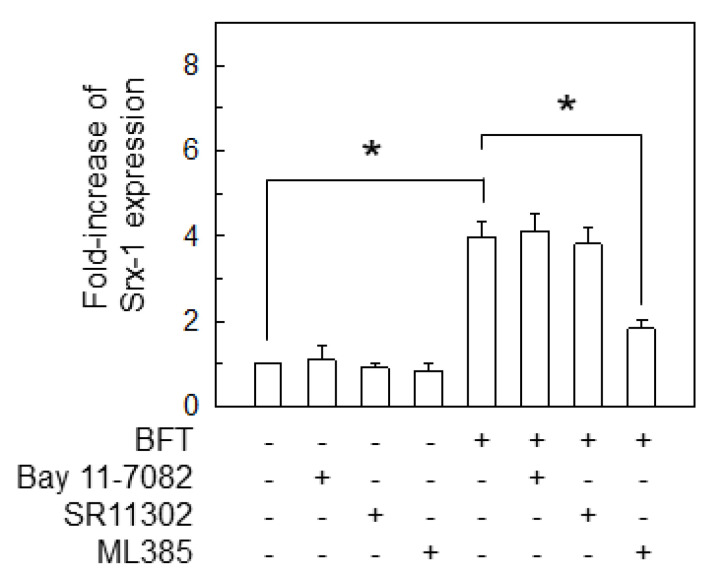
Inhibitory effects of Srx-1-expression by chemical inhibitors. CCD 841 CoN cells were combined with chemical inhibitors such as Bay 11–7082 (50 μM), SR11302 (10 μM) or ML385 (5 μM) for 1 h. BFT (300 ng/mL) was then added to each group for an additional 18 h. Analysis of protein-expression of Srx-1 was determined by ELISA. Data were obtained through five independent experiments and expressed as mean ± SEM. The asterisk indicates that the *p*-value is less than 0.05 compared to BFT alone.

To confirm these results, CCD 841 CoN cells were incubated with chemical inhibitors such as Bay 11–7082 (NF-κB inhibitor), SR11302 (AP-1 inhibitor) or ML385 (Nrf2 inhibitor), followed by BFT treatment. Results showed that Bay 11–7082 and SR11302 did not influence Srx-1-expression compared with no pretreatment under BFT-stimulated conditions. However, ML385 and BFT combined treatment attenuated BFT-induced Srx-1-expression (Figure 5).

### 2.4. Activation of p38 Signals Is Essential to Induce Srx-1-Expression in BFT-Stimulated IECs

**Figure 6:**
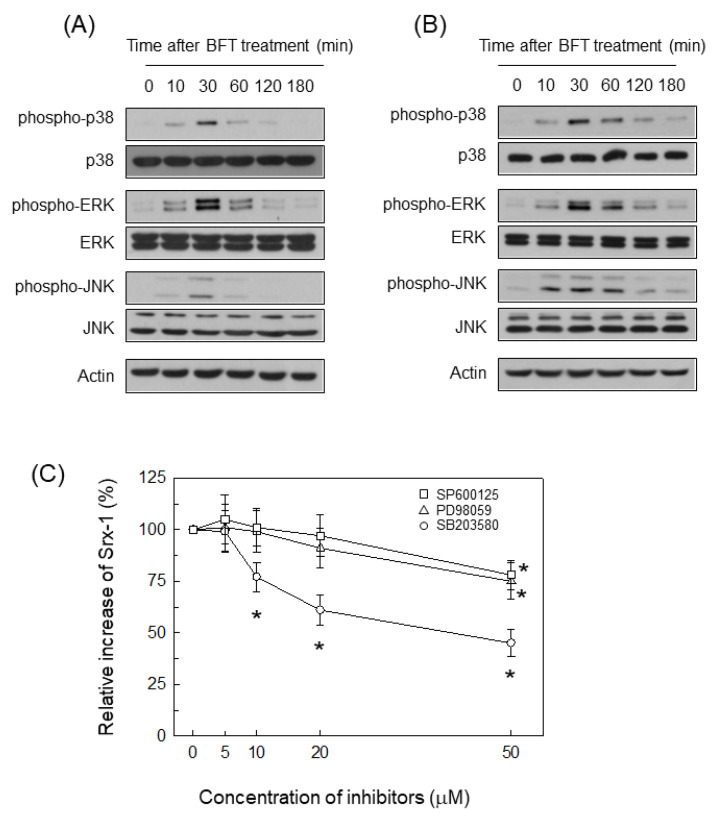
Relationship between chemical inhibition and Srx-1-expression. (**A**,**B**) BFT (300 ng/mL) was added to CCD 841 CoN cells (**A**) and HCT 116 cells (**B**) for the indicated periods of time. Protein-expression of MAPK signals such as phospho-p38, p38, phospho-ERK1/2, ERK1/2, phospho-JNK and JNK was examined by western blot. All images in (**A**,**B**) are representative of more than three independent experiments; (**C**) CCD 841 CoN cells were combined with SP600125, PD98059 or SB203580 for 30 min. BFT (300 ng/mL) was then added to each group for 18 h. Analysis of protein expression of Srx-1 was determined by ELISA. Data were obtained through five independent experiments and expressed as mean% increase relative to unstimulated controls ± SEM. The asterisk indicates that the *p*-value is less than 0.05 compared to BFT alone.

BFT increased phosphorylated forms of ERK1/2, p38 and JNK proteins in HCT 116 cells (Figure 6A). Similar results were noted in CCD 841 CoN cells treated with BFT (Figure 6B). We next assessed if treatment with chemical inhibitors may influence the BFT-induced expression of Srx-1. In this experiment, chemical inhibitors were used for suppressing MAPK activities [19,26]. Pretreatment of normal epithelial CCD 841 CoN cells with PD98059 (≥50 μM), SB203580 (≥10 μM) or SP600125 (≥50 μM) significantly reduced Srx-1-expression (Figure 6C).

**Figure 7:**
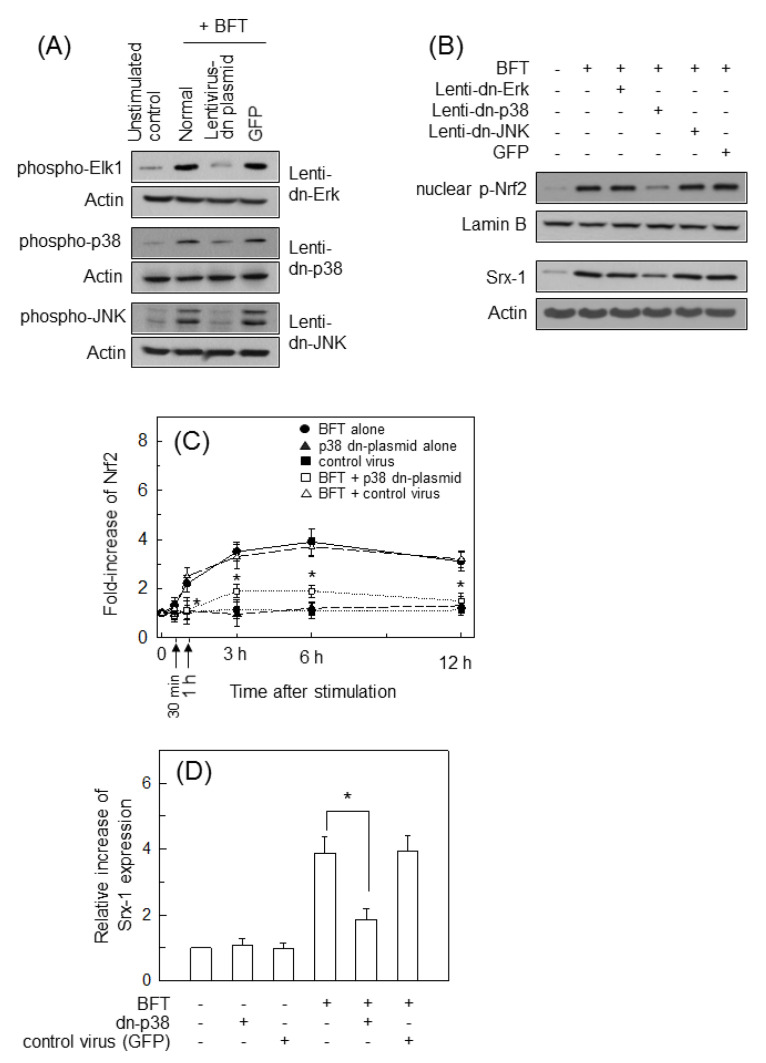
Relationship between Srx-1 expression and MAPK-suppression in BFT-exposed HCT 116 cells. (**A**) HCT 116 cells were transfected with lentiviruses containing either a dominant-negative or a control plasmid (GFP). BFT (300 ng/mL) was added to each group for 30 min. Protein expression was determined by western blot; (**B**) BFT (300 ng/mL) was added to each group for 6 h (phospho-Nrf2, top panels) or 18 h (Srx-1, bottom panels). Protein expression was also determined by western blot. All images in (**A**,**B**) are representative of more than three independent experiments; (**C**) BFT (300 ng/mL) was added to each group for the indicated periods of time. Analysis of Nrf2 activity was assessed by ELISA. Data were obtained through five independent experiments and expressed as mean fold induction ± SEM. The asterisk indicates that the *p*-value is less than 0.05 compared to BFT alone; (**D**) BFT (300 ng/mL) was added to each group for 18 h. Analysis of protein expression of Srx-1 was assessed by ELISA. Data were obtained through five independent experiments and expressed as mean increase relative to unstimulated controls ± SEM. The asterisk indicates that the *p*-value is less than 0.05 compared to BFT alone.

To confirm these results, lentivirus-based transfection systems were used as previously described [19,25]. Phosphorylated forms of three MAPK proteins were suppressed to levels of control cells when HCT 116 cells were infected with lentiviruses containing each dominant-negative plasmid (Figure 7A). In these experimental systems, cells infected with p38-suppressing lentivirus showed reduced BFT-induced phospho-Nrf2 expression compared with untransfected cells (Figure 7B, top panels). However, transfection with dominant-negative Erk2 or dominant-negative JNK1 showed no difference in phospho-p65 expression compared with non-transfection controls under BFT-treated conditions. Transfection with dominant-negative p38 led to inhibiting Srx-1 expression compared with untransfected cells under BFT-exposed conditions (Figure 7B, bottom panels). In another experiment, Nrf2 activity and Srx-1 expression were measured using ELISA kits. Transfection with dominant-negative p38 meaningfully reduced Nrf2 activity under BFT-exposed conditions (Figure 7C). Moreover, the p38-suppressing lentivirus significantly inhibited Srx-1 expression following BFT stimulation compared with non-transfection controls (Figure 7D). These results propose that a signaling cascade including p38, Nrf2 and Srx-1 signals may be activated after BFT exposure to IECs.

### 2.5. Srx-1 Induction Is Connected with the Apoptosis in IECs Stimulated with BFT

**Figure 8:**
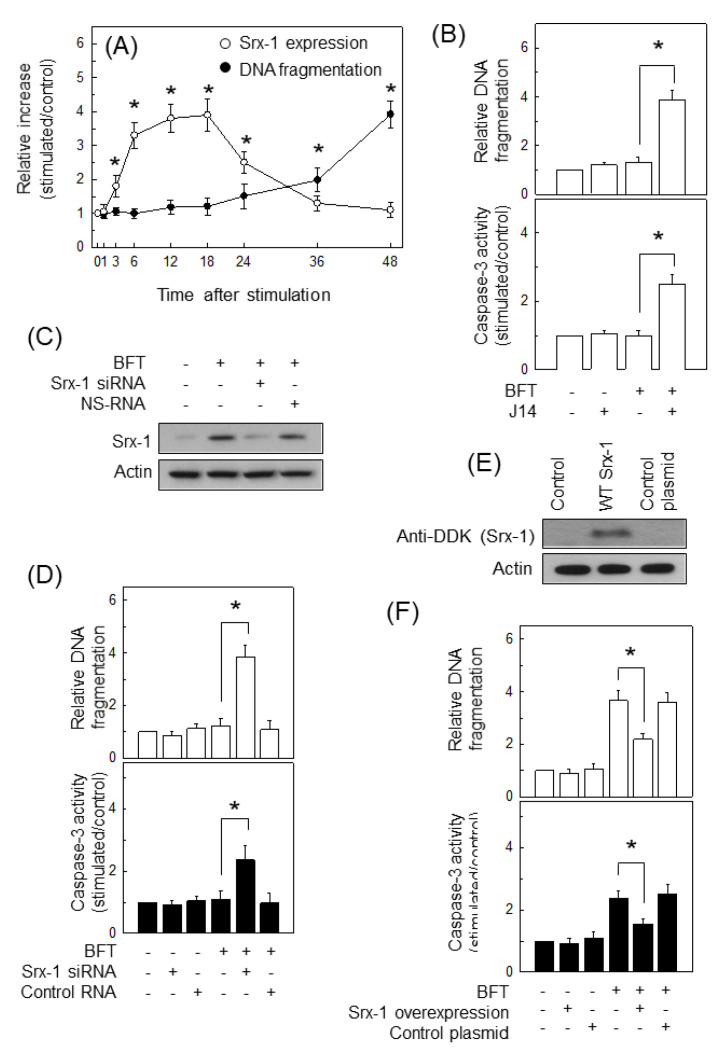
Relationship between Srx-1 expression and apoptosis. (**A**) After adding BFT (500 ng/mL) to CCD 841 CoN cells, DNA fragmentation and Srx-1 expression were measured at the indicated periods of time using each ELISA kit. Data were obtained through five independent experiments and expressed as mean increase relative to unstimulated controls ± SEM. The asterisk indicates that the *p*-value is less than 0.05; (**B**) CCD 841 CoN cells were combined with J14 (25 μM) for 18 h. BFT (500 ng/mL) was then added to each group for 12 h. DNA fragmentation and caspase-3 activity were measured by ELISA. Quantitative data analyses were performed with five independent experiments and expressed as mean increase relative to unstimulated controls ± SEM. The asterisk indicates that the *p*-value is less than 0.05; (**C**) HCT 116 cells were transfected with siRNA against Srx-1 or NS-RNA. BFT (500 ng/mL) was added to each group for 18 h. Protein expression was assessed by western blot; (**D**) Transfection was identical to (**C**). BFT (500 ng/mL) was added to each group for 12 h. Measuring DNA fragmentation and caspase-3 activity were identical to (**B**). Data were obtained through five independent experiments and expressed as mean increase relative to unstimulated controls ± SEM. The asterisk indicates that the *p*-value is less than 0.05; (**E**) Wild type (WT)-Srx-1-overexpressing plasmids were introduced into HCT 116 cells using a lentiviral system. The protein expression of Srx-1 was evaluated by western blot. All images in (**C**) and (**E**) are representative of more than three independent experiments; (**F**) Transfection was identical to (**E**). BFT (500 ng/mL) was added to each group for 48 h. Measuring DNA fragmentation and caspase-3 activity were identical to (**B**). Data were obtained through five independent experiments and expressed as mean increase relative to unstimulated controls ± SEM. The asterisk indicates that the *p*-value is less than 0.05.

Our previous studies demonstrated that treatment with 500 ng/mL of BFT was required for the induction of apoptosis in IECs [25,27]. Based on our previous results, we assessed the relationship between apoptosis and Srx-1 expression. Stimulation of CCD 841 CoN cells with BFT, as shown in Figure 8A, induced DNA fragmentation characteristic of apoptosis. DNA fragmentation was not found within 24 h post-stimulation in BFT-treated cells. Apoptosis was observed approximately 36 h post-stimulation, after which BFT-induced apoptosis increased. In contrast to apoptosis, a significant increase of Srx-1 expression was observed in the early period of stimulation. These results suggest that apoptosis is a relatively late epithelial cell response to BFT exposure compared to Srx-1 induction.

In the next experiment, we asked if Srx-1 upregulation is connected with the suppression of apoptosis in a relatively early period of BFT stimulation. Pretreatment of HCT 116 cells with Nrf2 inhibitor J14 significantly enhanced both caspase-3 activity and DNA fragmentation 12 h post-stimulation (Figure 8B). In another experiment, transfection of HCT 116 cells with Srx-1 siRNA suppressed the increased Srx-1 signal to control levels following BFT stimulation compared with untransfected cells (Figure 8C). In this experimental system, Srx-1 siRNA meaningfully enhanced the caspase-3 activity and the DNA fragmentation when apoptosis was not observed 12 h after stimulation with BFT (Figure 8D). To confirm these results, HCT 116 cells were infected with lentiviral vectors containing the overexpressing plasmids for Srx-1 (Figure 8E). Results showed that Srx-1 overexpression reduced the caspase-3 activity and the DNA fragmentation when apoptosis was apparently observed 48 h after stimulation with BFT (Figure 8F).

## 3. Discussion

Toxigenic *B. fragilis* is a non-invasive bacterium that secrets enterotoxin, which is responsible for diseases caused by bacterial infection [1,2,3,4]. Theoretically, BFT first contacts IECs and then provokes the inflammatory response in the gut. This study exhibits that exposure of IECs to BFT induces an increase in Srx-1 expression.

Increased expression of Srx-1 has been demonstrated in several cell types treated with stimulators. Srx-1 expression is upregulated in IECs; for example, Srx-1 was favorably expressed in poorly differentiated colorectal cancer cells and treatment with H_2_O_2_ increased Srx-1 protein expression in RKO, HCT 116 and Geo cells [28]. In addition, treatment with atmospheric pressure gas plasmas was shown to increase Srx-1 expression in HT-29 cells [29]. In the present study, we used HCT 116 cells and primary normal epithelial CCD 841 CoN cells. However, we did not find that BFT changed the expression of Srx-1 in other IEC lines such as RKO (ATCC CRL-2577) or HT-29 cells (ATCC HTB-38) (data not shown). Therefore, BFT-induced expression of Srx -1 seems to be expressed differently depending on the type of IECs. Nevertheless, our results indicate that Srx-1 upregulation may play an important role in BFT-associated pathogenesis.

Transcriptional factors including Nrf2, AP-1 and NF-κB control the mucosal cell response in the gut. We already demonstrated that BFT activates these transcription factors in IECs [4,7,19,23,24]. Several studies proved that Srx-1 expression is regulated by transcriptional factors in a variety of cells [20,21,22,30]. In the present study, Nrf2 activation in HCT 116 cells was assessed using immunoblot and Nrf2-DNA binding activity, and the specificity was analyzed by supershift and competition assays. This study revealed that suppression of Nrf2 activity using either transfection or chemical treatment led to a significant decrease of Srx-1 expression in BFT-exposed IECs. However, suppression of NF-κB or AP-1 activity did not significantly influence Srx-1 expression in BFT-treated cells. These results suggest that Nrf2 activation is closely related to Srx-1 upregulation in IECs contacted with BFT.

In the present study, we used rabbit polyclonal Ab against phospho-Nrf2, in which the Ab binds the phosphorylation site of Serine 40 in human Nrf2. Under resting state, Nrf2 is sequestered in the cytosol by a Keap1 homodimer which facilitates the ubiquitination and proteasomal degradation of Nrf2. In cells exposed to chemicals or oxidative stress, a conformational change in Keap1 mediated via its reactive cysteine residues results in the release of Nrf2 from one Keap1 molecule. Therefore, Nrf2 can no longer be ubiquitinated and degraded Keap1 becomes fully saturated with Nrf2, allowing newly synthesized Nrf2 to accumulate and translocate to the nucleus [31,32,33,34]. Thus, the activation of Nrf2 by protein kinase C can phosphorylate Serine 40 of Neh2 domain, which is essential for Keap1 and Nrf2 dissociation [31,32,33,34]. This dissociation promotes the translocation of Nrf2 into the nucleus [31]. In addition, a study showed that Nrf2 accumulated in the nucleus is in a phosphorylated state when Nrf2 signaling is activated [32]. In these experiments using phospho-Nrf2-specific Abs, identified proteins in the nucleus were phospho-Nrf2, not Nrf2 [34]. To confirm the activation of Nrf2, we performed another experiment regarding the Nrf2-DNA-binding using EMSA. Results showed that BFT enhanced the DNA-binding activity of Nrf2 in HCT 116 cells. Moreover, the specificity of Nrf2-DNA binding was confirmed by competition and inhibition assays. Therefore, we have estimated that Nrf2 accumulated in the nucleus may be in a phosphorylated state when BFT activates Nrf2 signaling.

MAPK signaling is known to be connected with Srx-1 expression. For example, Srx-1 was shown to enhance the survival of cardiac cells against oxidative stress through increased expression of the ERK signaling [35]. When mouse skin epithelial JB6 cells were combined treated with TPA and chemical inhibitors such as the ERK inhibitor PD98059 or the JNK inhibitor SP600125, TPA-induced Srx-1 expression was decreased [36]. However, there are no reports regarding the relationship between MAPK–Nrf2 activation and Srx-1 expression in BFT-exposed IECs. This study showed that p38 inhibitor SB203580 was superior to SP600125 or PD98059 in attenuating Srx-1 induction. Moreover, when p38 MAPK activity was suppressed using a lentivirus-based knockdown strategy, both Nrf2 activation and Srx-1 expression were significantly reduced in BFT-treated cells. These results propose that BFT may activate a signaling pathway including p38 MAPK–Nrf2 activation connected to the upregulation of Srx-1 in IECs. The p38 MAPK and Nrf2-dependent induction of Srx-1 may be a distinctive characteristic of BFT-exposed IECs. In the present study, we used a pharmacological dose of BFT to induce Srx-1 expression. Therefore, further research is needed to elucidate whether Srx-1 expression may be changed in IECs during ETBF infection in vivo.

Delayed apoptosis has been observed in BFT-exposed IECs [27,37]. These findings raise the possibility that the generation of signals for preventing apoptosis is evoked in BFT-treated IECs. In a series of experiments to demonstrate this hypothesis, we already reported that the enhanced expression of cellular inhibitor of apoptosis protein-2 attenuates the apoptosis in IECs [27]. In addition, BFT-induced heme oxygenase-1 (HO-1) upregulation is also closely connected with the suppression of apoptosis [37]. However, overexpression of HO-1 did not completely prevent apoptosis. Therefore, some other factors may work for cell protection in BFT-exposed IECs. In our study, when Srx-1 expression reached the baseline level in BFT-exposed IECs, apoptosis was noted 36 to 48 h post-stimulation with BFT. In addition, a strategy to exogenously increase Srx-1 expression resulted in a significant reduction of apoptosis in BFT-stimulated cells.

The upregulation of Srx-1 in the present study and HO-1 in the previous study [31] is a transient phenomenon in IECs exposed to BFT. In addition, apoptosis appears to be temporarily delayed [27,37]. Thus, there may be a purpose to temporarily suppress apoptosis in the early stages of ETBF infection. Considering the results that most of the inflammatory signals are expressed early during BFT stimulation, it is assumed that during the period when cells are protected from apoptosis, IECs in contact with BFT may be allowed to induce inflammatory responses. Moreover, delayed apoptotic cell death may be essential to the host infected with toxigenic *B. fragilis* because appropriate time is necessary for generating mediators or signals to avoid bacterial pathogenic effects. As proof of this hypothesis, endogenous antimicrobial factors, including hBD-2 [38] and lipocalin-2 [39], were increased in IECs stimulated with BFT. In addition, clinical illnesses of ETBF infection are self-limiting with transient colitis [1]. Considering these reports, endogenous antimicrobial factors in the intestinal mucosa of the bacteria-infected persons are likely to act as a countermeasure to properly control the response to BFT.

There is some weakness in this study. For example, we obtained the experimental results mainly using immunoblot assay. However, it is necessary to perform immunostaining or flow cytometry to confirm the results regarding Srx-1 level, NF-κB and Nrf2 activation. In addition, the rationale of investigation of the Srx-1 may be rather vague as there may be more important downstream effectors of BFT if global alterations of gene expressions are investigated. Therefore, research on this will be needed in the future; (B) *fragilis* strains causing intestinal secretion are named ETBF and their non-secretory strains are called nontoxigenic *B. fragilis* (NTBF) [2]. Therefore, an imbalance in microbiota composition with an excess of Bacteroides may have a detrimental effect although *B. fragilis* accounts for only 0.5% of the human colonic flora. Research into this imbalance may be required.

**Figure 9:**
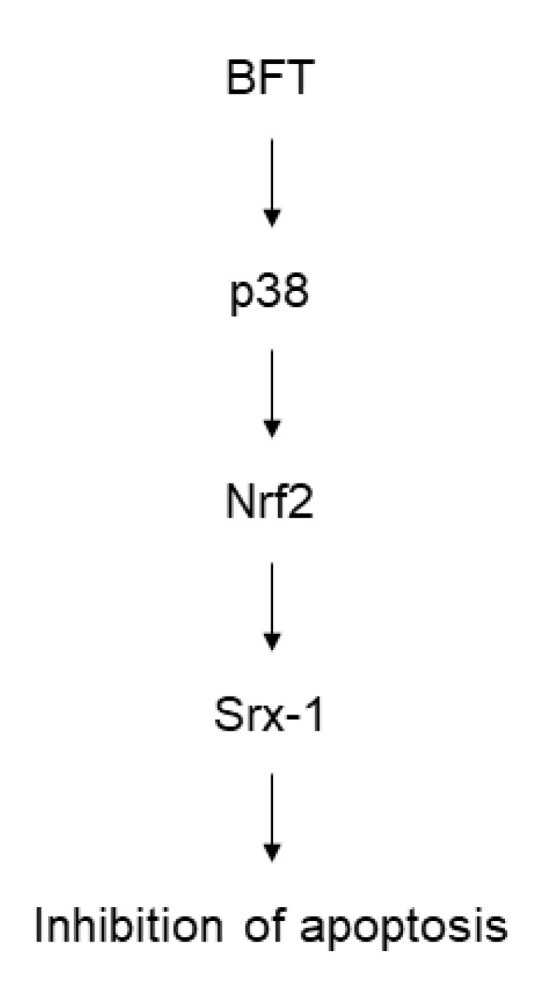
Schematic summary indicating BFT-induced signaling involved in Srx-1 induction and apoptotic inhibition in IECs.

In summary, we demonstrated that exposure of IECs to BFT resulted in the rapid activation of p38 MAPK signaling. Activated MAPK signals led to the induction of Srx-1 molecules via the activation of Nrf2 in IEC. The resulting upregulation of Srx-1 expression may regulate the apoptotic process in response to BFT stimulation (Figure 9).

## 4. Materials and Methods

### 4.1. Reagents

Antibiotics, phosphate-buffered saline (PBS) and TRIzol were achieved from GIBCO BRL (Gaithersburg, MD, USA). N-[4-[2,3-Dihydro-1-(2-methylbenzoyl)-1*H*-indol-5-yl]-5-methyl-2-thiazolyl]-1,3-benzodioxole-5-acetamide (ML385), bovine serum albumin (BSA), skim milk, Tween-20 and L-sulforaphane were obtained from Sigma Chemical Co. (St. Louis, MO, USA). Cell Signaling Technology, Inc. (Beverly, MA, USA) supported Abs against several molecules such as phospho-c-jun (Cat No. 9164), phospho-p65 (Cat No. 3033), phospho-IκBα (Cat No. 2859), phospho-p38 (Cat No. 9211), phospho-ERK1/2 (Cat No. 9101), phospho-JNK (Cat No. 9251), pan-p38 (Cat No. 8690), pan-ERK1/2 (p44/p42) (Cat No. 9102), pan-JNK (p54/p46) (Cat No. 9252). Santa Cruz Biotechnology (Santa Cruz, CA, USA) supported mouse primary Abs against actin (sc-47778) and lamin B (sc-374,015) and goat anti-mouse (sc-2005) and anti-rabbit (sc-2004) Abs conjugated to horseradish peroxidase. Bioss Antibodies, Inc. (Woburn, MA, USA) supported rabbit anti-phospho-Nrf2 (bs-2013R). Chemical inhibitors such as PD98059, SB203580, SP600125 and Bay 11-7085 were purchased from Calbiochem (La Jolla, CA, USA). J14 and SR11302 were purchased from MedChemExpress (Monmouth Junction, NJ, USA) and Tocris Bioscience (Bristol, UK), respectively.

### 4.2. Cell Cultures and Purification of BFT

HCT 116 cells (ATCC CCL-247, a human colonic epithelial cell line) were cultured in McCoy’s 5a medium supplemented with 10% fetal bovine serum (FBS, LPS-free) and antibiotics (100 μg/mL of streptomycin and 100 units/mL of penicillin) as described previously [8]. A normal colonic epithelial cell line CCD 841 CoN (ATCC CRL-1790) was grown in Eagle’s minimum essential medium with 10% FBS and 1-mM sodium pyruvate [8]. Before cell stimulation, the medium was removed, after which a fresh culture medium was added. After cells were incubated with BFT for the indicated periods, cells were washed with PBS and harvested using a cell scraper. Collected cells were transferred to e-tube and spin down for 1 min at 3000 rpm. Whole lysate proteins were extracted using PRO-PREP™ protein extraction solution (iNtRON, Seongnam-si, Gyeonggi-do, Republic of Korea) and nuclear proteins were extracted with NE-PER™ (Thermo Scientific, Waltham, MA, USA). The extraction procedure was conducted according to protocols supported by the individual manufacturers. BFT was purified from culture supernatants of a toxigenic *B. fragilis* strain (ATCC 43,858) as described previously [8,19,25,26,37].

### 4.3. Experiments of Transfection

For blocking activated signals such as Nrf2, AP-1, NF-κB and MAPKs, a lentivirus-based knockdown strategy was used as described previously [19,25,37]. In these experiments, the lentiviral systems used are identical to our previous studies [19,25,37] that were supported by the BioCore at the Institute of Biomedical Science (Seoul, Korea). Santa Cruz Biotechnology supported lentiviral vectors containing Nrf2 shRNA plasmid (human) and a control lentivirus. OriGene Technologies, Inc. (Rockville, MD, USA) supported human Srx-1 cDNA clone and kits for lentiviral construction. All experiments related to transfection were accomplished according to the protocols supported by the individual manufacturers [37].

Small interfering RNA (siRNA) against the Nrf2, c-jun, NF-κB p65 subunit and Srx-1 were designed as described previously [25,37]. Santa Cruz Biotechnology supported all siRNAs against the Nrf2, c-jun, p65 subunit, Srx-1 and a non-silencing siRNA (NS-RNA) as a negative control. Experiments were performed according to a protocol as described previously [8,19,25,37].

### 4.4. Western Blotting and ELISA

Immunoblot analysis for detecting expressed proteins was performed according to a protocol, as described previously [8,19,25]. After transfer procedure, blotted membranes were incubated in 5% (*w/v*) skim milk in Tris-buffered saline containing 0.1% Tween-20 (TBST) for 1 h for blocking. After that, the membranes were washed with TBST for 5 min and then incubated with primary Abs at 1:4000 dilution [Abs against phospho-c-jun, phospho-p65, phospho-IκBα, phospho-p38, phospho-ERK1/2, phospho-JNK, pan-p38, pan-ERK1/2 (p44/p42) and pan-JNK (p54/p46)] or 1:2000 dilution (Abs against Srx-1, phospho-Nrf2, actin and lamin B) in 5% (*w/v*) BSA in TBST and incubated overnight at 4 °C. The membranes were washed 3 times with 10 mL of TBST for 5 min each. After then, the membranes were incubated with secondary Abs (1:6000) in 5% (*w/v*) BSA in TBST for 1 h. Goat anti-mouse or anti-rabbit Abs conjugated to horseradish peroxidase were used as secondary Abs. The reaction bands were detected using ECL system (GenDepot, Katy, TX, USA) and X-ray film exposure.

The phospho-Elk1 proteins were detected using a p44/42 MAPK assay kit (Cell Signaling Technology) as described previously [19,25]. ELISA kits were used for measuring the protein levels of Srx-1 (MyBioSource, San Diego, CA, USA) and Nrf2 (Active Motif, Inc., Carlsbad, CA, USA). Each assay was carried out under protocols supported by the individual manufacturers [19,25].

### 4.5. Electrophoretic Mobility Shift Assay (EMSA)

The experiments regarding EMSA for Nrf2 were performed as we described previously [19,40]. For these experiments, a commercial kit (Promega, Madison, WI, USA) was used. Briefl, 5 µg of nuclear extract was incubated for 30 min at room temperature with γ32P-labeled oligonucleotide probes (Santa Cruz Biotechnology, 5′-TGG GGA ACC TGT GCT GAG TCA CTG GAG-3′). Nrf2 supershift and competition assay were also performed as previously described [19,40].

### 4.6. Analysis of Apoptosis

For determining DNA fragmentation, a Cell Death Detection ELISA^PLUS^ kit (Roche Diagnostics) was used as described previously [25]. Caspase-3 activity was measured using a commercially available kit (R&D Systems, Minneapolis, MN, USA). Each assay was performed according to protocols supported by the individual manufacturers [25].

### 4.7. Statistics

Data in the present study are described as the mean ± standard error of the mean (SEM) or the mean ± standard deviation (SD). Statistical analysis was evaluated by the Mann–Whitney t-test. A *p*-value less than 0.05 was considered statistically significant.
